# A human-centric fuzzy decision support system for medical diagnosis using fuzzy cognitive maps

**DOI:** 10.1038/s41598-026-51590-z

**Published:** 2026-05-18

**Authors:** Amr Zakaria

**Affiliations:** Department of Mathematics, Faculty of Education, Ain Shams University, Cairo, 11341 Egypt

**Keywords:** Computational biology and bioinformatics, Health care, Mathematics and computing

## Abstract

Medical decision support requires models that remain interpretable under uncertainty while still adapting to evolving data and expert knowledge. Fuzzy cognitive maps (FCMs) are attractive in this setting because they encode concept-level relations in a transparent graphical form. However, static expert-defined FCMs are often too rigid, whereas purely data-driven updates may weaken semantic consistency and drift away from clinically meaningful relations. This paper proposes a human-centric adaptive framework for fuzzy cognitive map-based medical decision support. The proposed Human-Centric Fuzzy Decision Support System (HCFDSS) combines data-driven weight adjustment with an explicit expert-correction operator, allowing the causal structure of the map to be refined without discarding domain knowledge. The method is formulated through a two-stage procedure: an inner reasoning loop updates concept activations for fixed weights, while an outer learning loop updates the weight matrix using a regularized objective together with bounded expert intervention. Under standard smoothness and boundedness assumptions, we establish sufficient conditions for stability of the inner reasoning dynamics and convergence of the outer weight-update operator. A numerical illustration shows how expert feedback can revise clinically important edges and alter the final diagnostic activation in an interpretable way. In addition, the paper reports a reproducible empirical evaluation involving synthetic medical scenarios and three public medical benchmark datasets from the UCI Machine Learning Repository, together with sensitivity analysis for expert intervention, preprocessing validation, robustness checks under missingness and imbalance, and expanded comparisons with mainstream machine-learning baselines. The proposed HCFDSS is positioned not as a replacement for high-capacity black-box predictors, but as a transparent and editable decision-support framework in which clinicians can inspect, question, and refine the learned relations. The main contribution of the present work is therefore methodological and theoretical, while the empirical evaluation clarifies the predictive–interpretability trade-off of the proposed framework under reproducible benchmark settings.

## Introduction

Medical decision-making is shaped by uncertainty, incomplete information, heterogeneous patient profiles, and linguistic clinical judgments that are rarely captured well by rigid binary logic. Fuzzy systems remain attractive in this context because they allow gradual membership, soft thresholds, and concept-level reasoning, all of which are naturally aligned with the way clinicians often describe risk, severity, and progression. Among such models, fuzzy cognitive maps (FCMs) are especially appealing because they encode interactions among clinical concepts in a graphical and interpretable form^1–4^.

In a classical FCM, concepts represent symptoms, signs, laboratory indicators, or diagnostic hypotheses, and weighted directed edges encode positive or negative influence among them. This makes FCMs suitable for medical decision support, where understanding *why* a model suggests a diagnosis is often as important as the prediction itself^5–9^. Static expert-defined FCMs, however, face a practical limitation: once the initial weights are fixed, the model may struggle to adapt when patient populations, diagnostic protocols, or the underlying clinical understanding evolve.

To overcome this rigidity, a substantial body of work has proposed adaptive FCM learning rules based on Hebbian updates, gradient-based optimization, evolutionary procedures, and hybrid strategies that combine data-driven learning with prior knowledge^2,4,10^. These developments have improved flexibility, but they have also exposed an important conceptual difficulty. When an FCM is learned primarily from data, the resulting weights may reflect statistical association rather than clinically defensible causal influence. In other words, a learned edge may improve predictive fit while no longer retaining the semantic meaning that motivated the original map. This issue is closely related to the broader causality-versus-correlation concern discussed in the recent FCM and interpretable-AI literature^11–14^.

For this reason, the central challenge is not merely to make FCMs adaptive, but to make them adaptive in a way that remains inspectable, editable, and clinically coherent. Human-in-the-loop learning offers a natural response to this challenge: expert knowledge is not used only at initialization, but may also guide the evolution of the model during learning. Still, existing hybrid approaches vary considerably in how expert intervention is encoded, when it is applied, and whether it is coupled with a clearly stated stability analysis. In particular, many papers motivate expert-informed adaptation in intuitive terms, while leaving the correction mechanism, disagreement handling, or convergence assumptions only partially specified.

The present work studies a human-centric adaptive FCM framework for medical decision support in which expert intervention is introduced through an explicit correction operator inside the weight-update process. The contribution is intentionally modest and specific. We do not claim to solve causal discovery from observational data, nor do we claim that expert-guided FCMs are entirely new. Instead, we focus on three concrete aims:

1. To formulate a transparent update mechanism that combines gradient-based adaptation with bounded expert correction;
2. To separate the reasoning dynamics from the learning dynamics, thereby enabling a cleaner stability analysis;
3. To specify and report a reproducible empirical evaluation for the resulting framework using synthetic medical scenarios and three public medical benchmark datasets, with particular attention to interpretability, sensitivity to expert intervention, preprocessing validation, and robustness under imperfect data conditions.

A second motivation for this study comes from the practical tension between interpretability and predictive flexibility. Highly expressive models, including modern deep architectures, can achieve impressive performance in disease-specific medical tasks such as diabetic retinopathy grading and brain-tumor lesion classification^15–17^. However, such models are often difficult to interrogate at the concept level. By contrast, FCM-based models provide a structured representation in which nodes and edges remain understandable to human experts. The aim of the present framework is therefore not to compete with all black-box predictors on raw predictive power, but to support settings where clinicians benefit from inspecting the learned structure, modifying selected relations, and observing how those modifications change the final diagnostic state. In this sense, the proposed HCFDSS is closer to explainable and concept-level medical decision support than to purely predictive black-box diagnosis.

The methodology developed here is built around a two-stage view of adaptation. For fixed weights, concept activations evolve according to a standard bounded reasoning rule. After the inner reasoning process stabilizes, the weight matrix is updated through a regularized learning step and then corrected by an expert-guided operator acting on clinically reviewed edges. This separation avoids conflating time-varying reasoning dynamics with the outer learning dynamics and leads to a more defensible theoretical treatment than a fully coupled non-autonomous analysis.

The paper is organized as follows. Section “Mathematical preliminaries” reviews the mathematical preliminaries on fuzzy sets and fuzzy cognitive maps, and briefly recalls the main dynamic behaviors relevant to the present study. Section “Proposed human-centric fuzzy decision support system” introduces the proposed Human-Centric Fuzzy Decision Support System and gives the full training and inference procedure. Section “Numerical illustration” presents a numerical illustration showing how expert feedback modifies the map and the final diagnosis. Section “Theoretical analysis” develops a stability and convergence analysis under explicit assumptions. Section “Evaluation protocol and benchmark design” describes the experimental design, evaluation metrics, and comparative protocol. Section “Discussion” discusses interpretability, robustness, limitations, and practical implications. Section “Conclusion” concludes the paper.

## Mathematical preliminaries

This section briefly recalls the basic ingredients used in the proposed framework. We first review fuzzy sets as a mechanism for representing gradual membership, and then summarize fuzzy cognitive maps as concept-level dynamic models. We also clarify the dynamic regimes relevant to the present work, since the proposed method is designed to operate in a stable fixed-point setting rather than to exploit oscillatory behavior.

### Fuzzy sets

Let $X \subset \mathbb {R}$ denote the universe of discourse. Following Zadeh’s foundational formulation of fuzzy sets^18^, a fuzzy set *A* on *X* is defined by its membership function $\mu _A: X \rightarrow [0,1]$, and is written as

$$\begin{aligned} \begin{aligned} A = \{(x,\mu _A(x)) : x \in X\}. \end{aligned} \end{aligned}$$

The value $\mu _A(x)$ measures the degree to which *x* belongs to the fuzzy concept represented by *A*.

In medical decision support, fuzzy sets are useful because many clinically meaningful descriptors do not admit crisp boundaries. Terms such as *high blood pressure*, *moderate pain*, or *elevated glucose* are often interpreted gradually rather than categorically. A simple example is the linguistic concept *High Blood Pressure*, which may be modeled by a piecewise linear or Gaussian membership function. In the present work, Gaussian membership functions are used in the experimental section because they provide smooth transitions and are convenient for continuous optimization.

### Fuzzy cognitive maps

An FCM is a weighted directed graph

$$\begin{aligned} \begin{aligned} G=(C,E), \end{aligned} \end{aligned}$$

where $C=\{C_1,\dots ,C_n\}$ is the set of concepts and *E* is the set of directed edges. Each edge from $C_i$ to $C_j$ is associated with a weight $w_{ij} \in [-1,1]$, where positive values indicate promoting influence, negative values indicate inhibiting influence, and zero indicates no direct influence.

At iteration *t*, the activation state of the map is represented by

$$\begin{aligned} \begin{aligned} A^{(t)} = \big (A_1^{(t)},\dots ,A_n^{(t)}\big ) \in [0,1]^n. \end{aligned} \end{aligned}$$

Given a weight matrix $W=[w_{ij}]$, the standard bounded reasoning rule updates the activations by

1 $$\begin{aligned} A^{(t+1)} = f\!\left( A^{(t)}W\right) , \end{aligned}$$

where $f:[0,\infty ) \rightarrow [0,1]$ is applied componentwise. In this paper, *f* will typically denote a sigmoid activation,

$$\begin{aligned} \begin{aligned} f(x)=\frac{1}{1+e^{-\lambda x}}, \end{aligned} \end{aligned}$$

with steepness parameter $\lambda >0$.

The fixed-point relation associated with (1) is

$$\begin{aligned} \begin{aligned} A^* = f(A^*W). \end{aligned} \end{aligned}$$

In medical interpretation, the equilibrium vector $A^*$ represents a stable concept profile induced by the current causal structure encoded in *W*.

### Dynamic behavior of FCMs

Depending on the reasoning rule, activation function, and weight structure, FCMs may exhibit different dynamic regimes, including convergence to fixed points, convergence to periodic or limit-cycle behavior in some formulations, or unstable behavior when feedback becomes too strong. The present study is concerned with the fixed-point regime, because stable equilibrium states are the most natural for interpretable diagnostic decision support; convergence behavior of sigmoid FCMs has also been studied in related work^19^. Our theoretical analysis therefore imposes conditions that make the inner reasoning operator contractive for fixed weights.

### Notation used in the proposed method

For convenience, we summarize the main notation used in the sequel:

- $A^{(k,m)}$: activation vector at inner iteration *m* during outer iteration *k*;
- $W^{(k)}$: weight matrix at outer iteration *k*;
- *f*: componentwise activation function;
- $\lambda$: sigmoid steepness parameter;
- *J*(*W*): regularized learning objective for the weight matrix;
- $\eta$: data-driven learning step size;
- $\alpha _k$: expert-correction coefficient at outer iteration *k*;
- $\Omega =[-1,1]^{n\times n}$: admissible weight domain;
- $\tau$: classification threshold used on the final disease concept;
- $T_{\max }$: maximum number of inner reasoning iterations;
- $\varepsilon$: inner stopping tolerance.

The next section introduces the proposed human-centric adaptive framework and explains how the reasoning and weight-update stages are coupled.

## Proposed human-centric fuzzy decision support system

This section presents the proposed Human-Centric Fuzzy Decision Support System (HCFDSS). The method combines fuzzy concept modeling, FCM-based reasoning, data-driven weight learning, and explicit expert correction. To avoid ambiguity, we separate the model into an *inner reasoning stage*, where concept activations evolve for fixed weights, and an *outer learning stage*, where the weights are updated after the reasoning stage stabilizes.

### Overview of the framework

For each patient sample, the HCFDSS pipeline consists of the following steps:

1. Raw clinical variables are normalized;
2. Each variable is mapped to fuzzy linguistic concepts through membership functions;
3. An initial FCM topology and weight matrix are specified from prior knowledge;
4. The activation vector is iteratively updated for fixed weights;
5. After the inner reasoning loop stabilizes, the weight matrix is updated using a regularized objective;
6. Expert feedback is incorporated through a bounded correction operator acting on selected edges;
7. The final disease-related concept activation is converted into a diagnostic decision.

This structure allows the reasoning process to remain interpretable while still permitting controlled adaptation of the causal relations.

### Normalization and fuzzification

Let $x=(x_1,\dots ,x_d)$ denote the raw clinical feature vector. For each feature $x_j$, min–max normalization is performed on the training set:

2 $$\begin{aligned} \tilde{x}_j = \frac{x_j - x_j^{\min }}{x_j^{\max }-x_j^{\min }}, \end{aligned}$$

where $x_j^{\min }$ and $x_j^{\max }$ are computed from the training partition only and then reused for validation and test data.

Each normalized feature is represented by three Gaussian membership functions corresponding to the linguistic labels *Low*, *Medium*, and *High*:

3 $$\begin{aligned} \mu _{j,r}(z) = \exp \!\left( -\frac{(z-c_{j,r})^2}{2\sigma _{j,r}^2}\right) , \qquad r\in \{1,2,3\}. \end{aligned}$$

Here $c_{j,r}$ and $\sigma _{j,r}$ denote the center and spread of the *r*-th membership function for feature *j*. In the experiments, the initial values of these parameters are estimated from training data and then checked for semantic plausibility, so that the induced concepts remain clinically meaningful.

For transparency, the membership parameters used in the experiments are reported later in Table 2.

### Initial FCM topology and weight matrix

Let $C=\{C_1,\dots ,C_n\}$ denote the concept set. In the medical setting, these concepts may include symptoms, laboratory indicators, intermediate risk factors, and one or more diagnostic concepts. The initial topology is encoded by an adjacency mask

$$\begin{aligned} \begin{aligned} M=[m_{ij}] \in \{0,1\}^{n\times n}, \end{aligned} \end{aligned}$$

where $m_{ij}=1$ indicates that the edge from $C_i$ to $C_j$ is permitted by prior knowledge, and $m_{ij}=0$ forbids that edge. The initial weight matrix $W^{(0)}=[w_{ij}^{(0)}]$ satisfies

$$\begin{aligned} \begin{aligned} w_{ij}^{(0)} = 0 \qquad \text {whenever } m_{ij}=0, \end{aligned} \end{aligned}$$

and $$w_{ij}^{(0)} \in [-1,1]$$ otherwise.

In the present framework, the topology mask is kept fixed during training, while only the permitted weights are updated. This choice preserves semantic interpretability and prevents the learning stage from inventing clinically unsupported edges.

### Inner reasoning dynamics

For a fixed outer iteration *k* and fixed weight matrix $W^{(k)}$, the activation dynamics are computed through the inner recursion

4 $$\begin{aligned} A^{(k,m+1)} = f\!\left( A^{(k,m)}W^{(k)}\right) , \qquad m=0,1,2,\dots , \end{aligned}$$

where *f* is applied componentwise. The inner loop is stopped when either

5 $$\begin{aligned} \Vert A^{(k,m+1)}-A^{(k,m)}\Vert \le \varepsilon \end{aligned}$$

or the maximum number of iterations $T_{\max }$ is reached. The stabilized activation vector is denoted by

$$\begin{aligned} \begin{aligned} A^{(k,*)}. \end{aligned} \end{aligned}$$

For classification tasks involving a designated disease concept $C_d$, the model output is taken to be

$$\begin{aligned} \begin{aligned} \hat{y}^{(k)} = A_d^{(k,*)}. \end{aligned} \end{aligned}$$

A binary diagnosis is then obtained by thresholding:

6 $$\begin{aligned} \widehat{\textrm{class}}= {\left\{ \begin{array}{ll} 1, & \hat{y}^{(k)} \ge \tau ,\\ 0, & \hat{y}^{(k)} < \tau . \end{array}\right. } \end{aligned}$$

In the experimental section, the threshold $\tau$ should be selected on the validation set rather than fixed arbitrarily.

### Regularized learning objective

Let $\{(x^{(s)},y^{(s)})\}_{s=1}^N$ denote the training samples and labels, and let $\hat{y}^{(s)}(W)$ be the final disease activation produced by the inner reasoning loop for sample *s* under weight matrix *W*. We define the regularized objective

7 $$\begin{aligned} J(W)=\frac{1}{N}\sum _{s=1}^N \ell \!\left( \hat{y}^{(s)}(W),y^{(s)}\right) +\beta \Vert W\Vert _F^2, \end{aligned}$$

where $\ell$ denotes the classification loss and $\beta >0$ is a regularization parameter. For binary classification, one convenient choice is the cross-entropy loss

8 $$\begin{aligned} \ell (\hat{y},y)= -y\log (\hat{y})-(1-y)\log (1-\hat{y}). \end{aligned}$$

To address class imbalance or asymmetric clinical risk, a cost-sensitive version may be used:

9 $$\begin{aligned} J_{\textrm{cost}}(W)= \frac{1}{N}\sum _{s=1}^N \omega _{y^{(s)}}\, \ell \!\left( \hat{y}^{(s)}(W),y^{(s)}\right) +\beta \Vert W\Vert _F^2, \end{aligned}$$

where $\omega _0,\omega _1>0$ are class-dependent penalties.

### Gradient-based update and practical stabilization

The gradient term is computed through the chain rule:

10 $$\begin{aligned} \nabla J(W) = \frac{1}{N}\sum _{s=1}^N \frac{\partial \ell \!\left( \hat{y}^{(s)}(W),y^{(s)}\right) }{\partial \hat{y}^{(s)}} \frac{\partial \hat{y}^{(s)}(W)}{\partial W} +2\beta W. \end{aligned}$$

In practice, $\partial \hat{y}^{(s)}(W)/\partial W$ is evaluated through truncated backpropagation over the inner reasoning iterations up to convergence tolerance or to $T_{\max }$, in line with recurrence-aware training ideas for cognitive-network models^20^. Since repeated application of a sigmoid may produce small derivatives, the implementation uses the following stabilizing choices:

- Bounded $\lambda$ values for the sigmoid function;
- Gradient clipping;
- Regularization through $$\beta \Vert W\Vert _F^2$$;
- Fixed stopping criteria for the inner loop.

These choices do not eliminate all optimization difficulties, but they reduce numerical instability and make the learning process more reproducible.

The data-driven candidate update is defined by

11 $$\begin{aligned} \widetilde{W}^{(k+1)} = \operatorname {Proj}_{\Omega }\!\left( W^{(k)}-\eta \nabla J(W^{(k)}) \right) , \qquad \Omega =[-1,1]^{n\times n}, \end{aligned}$$

where $\operatorname {Proj}_{\Omega }$ denotes the projection onto the admissible weight domain.

### Expert correction operator

At outer iteration *k*, the expert reviews a subset of clinically relevant edges

$$\begin{aligned} \begin{aligned} \mathcal {S}_k \subseteq \{(i,j): m_{ij}=1\}. \end{aligned} \end{aligned}$$

An expert-proposed matrix $W_E^{(k)}$ is then formed as follows:

$$\begin{aligned} \begin{aligned} (W_E^{(k)})_{ij} = {\left\{ \begin{array}{ll} \bar{w}_{ij}^{(k)}, & (i,j)\in \mathcal {S}_k, \\ \widetilde{w}_{ij}^{(k+1)}, & (i,j)\notin \mathcal {S}_k, \end{array}\right. } \end{aligned} \end{aligned}$$

where $\bar{w}_{ij}^{(k)}$ is the expert-recommended value for the reviewed edge.

The final updated matrix is obtained by convex blending:

12 $$\begin{aligned} W^{(k+1)} = \operatorname {Proj}_{\Omega }\!\left( (1-\alpha _k)\widetilde{W}^{(k+1)} + \alpha _k W_E^{(k)} \right) , \qquad 0\le \alpha _k < 1. \end{aligned}$$

Thus, disagreement between the data-driven update and the expert recommendation is handled through a controlled interpolation rather than a hard overwrite. This is important both conceptually and numerically: it preserves expert influence without making the learning step discontinuous.

### Complete training procedure

The complete procedure is summarized below.

**Algorithm 1:**
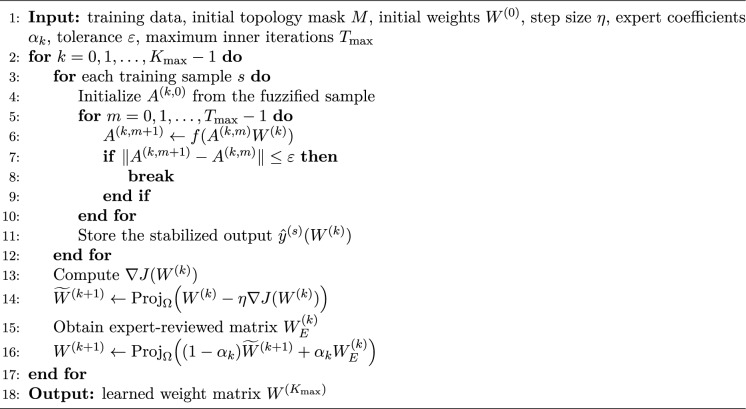
Training procedure for HCFDSS

## Numerical illustration

We now illustrate the behavior of the proposed framework on a simple three-concept cardiovascular example. The aim is not to claim medical validation from this toy setting, but to show, in a transparent way, how the expert-correction mechanism alters the learned structure and the final diagnostic activation.

Consider the concept set

$$\begin{aligned} \begin{aligned} C_1=\text {High Blood Pressure},\qquad C_2=\text {Chest Pain},\qquad C_3=\text {Heart Disease}. \end{aligned} \end{aligned}$$

The initial weight matrix is

13 $$\begin{aligned} W^{(0)}= \begin{bmatrix} 0 & 0.30 & 0.40\\ 0 & 0 & 0.60\\ 0 & 0 & 0 \end{bmatrix}. \end{aligned}$$

This encoding reflects the assumptions that elevated blood pressure promotes chest pain and heart disease, while chest pain also promotes the final diagnostic concept.

Suppose the initial activation vector for a patient is

$$\begin{aligned} \begin{aligned} A^{(0,0)}=[0.80,\,0.60,\,0.00]. \end{aligned} \end{aligned}$$

Using the sigmoid activation function with $\lambda =1$, the first reasoning step yields

$$\begin{aligned} \begin{aligned} A^{(0,1)}=f(A^{(0,0)}W^{(0)}) = f([0,\,0.24,\,0.68]) \approx [0.50,\,0.56,\,0.66]. \end{aligned} \end{aligned}$$

Thus, after one propagation step, the heart-disease concept already receives a substantial activation.

Assume that the treating physician reviews the edge from chest pain to heart disease and judges that the value $w_{23}=0.60$ underestimates the true clinical relation. The expert recommends a revised value of 0.80 for this edge. For illustration, take $\alpha _0=0.30$ and assume that the gradient contribution for this specific update is small relative to the expert correction. Then the expert-guided update becomes

$$\begin{aligned} \begin{aligned} w_{23}^{(1)} = (1-\alpha _0)\cdot 0.60+\alpha _0\cdot 0.80 = 0.66. \end{aligned} \end{aligned}$$

Hence the revised weight matrix is

14 $$\begin{aligned} W^{(1)}= \begin{bmatrix} 0 & 0.30 & 0.40\\ 0 & 0 & 0.66\\ 0 & 0 & 0 \end{bmatrix}. \end{aligned}$$

Running the reasoning rule again with the updated matrix gives

$$\begin{aligned} \begin{aligned} A^{(1,1)}=f(A^{(0,1)}W^{(1)}) = f([0,\,0.15,\,0.79]) \approx [0.50,\,0.54,\,0.69]. \end{aligned} \end{aligned}$$

Therefore, the final activation of the disease concept increases from approximately 0.66 to 0.69 after the expert correction.

If the operational threshold is chosen as $\tau =0.60$ for this illustration, the final state corresponds to a positive classification of the disease concept. In the experimental section, however, $\tau$ should be selected by validation rather than fixed by illustration.

**Fig. 1:**
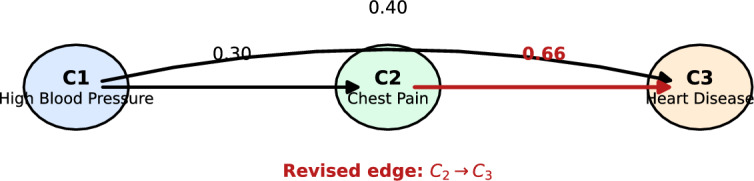
Three-concept FCM used in the numerical illustration. The edge $C_2 \rightarrow C_3$ is revised by expert intervention.

The example highlights the intended role of expert intervention in the HCFDSS framework. The correction is local, interpretable, and easy to justify clinically; at the same time, it changes the final decision through a transparent propagation mechanism rather than through a hidden latent representation. This is precisely the type of behavior sought in concept-level medical decision support (Fig. 1).

## Theoretical analysis

The goal of this section is to provide a mathematically defensible analysis of the proposed adaptive framework. Rather than treating the reasoning and learning dynamics as a single non-autonomous map, we analyze them in two stages. First, we study the inner activation dynamics for a fixed weight matrix. Second, we analyze the outer weight-update operator. This separation leads to assumptions and conclusions that are easier to justify and interpret.

### Assumptions

Throughout this section, $\Vert \cdot \Vert$ denotes a fixed operator-compatible norm on vectors and matrices.

- (A1) The activation function $f:\mathbb {R}\rightarrow [0,1]$ is Lipschitz continuous with constant $L_f$, and is applied componentwise to vectors.
- (A2) There exists $\kappa >0$ such that
  $$\begin{aligned} \begin{aligned} \Vert W\Vert \le \kappa < \frac{1}{L_f} \end{aligned} \end{aligned}$$
  for all weight matrices considered in the inner reasoning stage.
- (A3) Objective $J:\Omega \rightarrow \mathbb {R}$ is continuously differentiable on $\Omega =[-1,1]^{n\times n}$, its gradient is $L_J$-Lipschitz continuous, and *J* is $\mu$-strongly convex on $\Omega$ for some $\mu >0$.
- (A4) The expert matrix $W_E$ is bounded and belongs to $\Omega$.

Assumption (A3) is stronger than what may hold in every empirical implementation, but it is standard in contraction-based analyses of first-order methods and allows us to state a clear convergence result for the outer update.

The purpose of these assumptions is to provide a sufficient-condition analysis for the proposed two-stage training schedule rather than a universal convergence statement for all adaptive FCM variants.

### Inner fixed-weight reasoning

For a fixed matrix $W\in \Omega$, define the operator

$$\begin{aligned} \begin{aligned} T_W(A)=f(AW), \qquad A\in [0,1]^n. \end{aligned} \end{aligned}$$

#### Proposition 5.3

*Under assumptions (A1)–(A2), the map *$T_W$ *is a contraction on *$[0,1]^n$* for every admissible fixed weight matrix W. Consequently, for each such W, there exists a unique fixed point *$A^*(W)\in [0,1]^n$* satisfying*

$$\begin{aligned} \begin{aligned} A^*(W)=f(A^*(W)W). \end{aligned} \end{aligned}$$

*Moreover, for any initial state *$A^{(0)}\in [0,1]^n$*, the recursion*

$$\begin{aligned} \begin{aligned} A^{(m+1)}=T_W(A^{(m)}) \end{aligned} \end{aligned}$$

*converges to *$$A^*(W)$$.

#### Proof

Let $A,B\in [0,1]^n$. Using the Lipschitz continuity of *f* and the submultiplicativity of the chosen norm, we obtain

$$\begin{aligned} \begin{aligned} \Vert T_W(A)-T_W(B)\Vert = \Vert f(AW)-f(BW)\Vert \le L_f \Vert (A-B)W\Vert \le L_f \Vert W\Vert \,\Vert A-B\Vert . \end{aligned} \end{aligned}$$

By assumption, $L_f\Vert W\Vert \le L_f\kappa <1$, hence $T_W$ is a contraction. The Banach fixed-point theorem then gives the existence and uniqueness of a fixed point $A^*(W)$, and the iterates converge to that fixed point for every initial condition. $\square$

#### Remark 5.5

Proposition 5.1 formalizes the fixed-point regime considered in this paper. It does not claim to characterize all possible FCM dynamics, nor does it exclude the possibility that other reasoning schemes may be preferable in tasks where multiple attractors or more elaborate scenario behavior is desired.

### Outer weight update

We now analyze the weight update operator. Define

$$\begin{aligned} \begin{aligned} G(W)=W-\eta \nabla J(W), \end{aligned} \end{aligned}$$

and let

$$\begin{aligned} \begin{aligned} F(W)=\operatorname {Proj}_{\Omega }\!\left( (1-\alpha )G(W)+\alpha W_E\right) , \qquad 0\le \alpha <1. \end{aligned} \end{aligned}$$

#### Proposition 5.1

*Assume (A3)–(A4) and choose the step size *$\eta$* so that*

$$\begin{aligned} \begin{aligned} 0<\eta <\frac{2}{L_J+\mu }. \end{aligned} \end{aligned}$$

*Then the gradient map G is a contraction on *$\Omega$* with contraction factor*

$$\begin{aligned} \begin{aligned} q=\max \{|1-\eta \mu |,\;|1-\eta L_J|\}<1. \end{aligned} \end{aligned}$$

*Consequently, the expert-corrected map F is also a contraction on *$\Omega$* with factor at most *$(1-\alpha )q$*, and therefore admits a unique fixed point *$W^*\in \Omega$.

#### Proof

For $\mu$-strongly convex and $L_J$-smooth objectives, the gradient step $G(W)=W-\eta \nabla J(W)$ is contractive whenever $0<\eta <2/(L_J+\mu )$, with factor

$$\begin{aligned} \begin{aligned} q=\max \{|1-\eta \mu |,\;|1-\eta L_J|\}<1. \end{aligned} \end{aligned}$$

Since the Euclidean projection onto a closed convex set is nonexpansive, we have for all $W,V\in \Omega$,

$$\begin{aligned} \Vert F(W)-F(V)\Vert&\le \Vert (1-\alpha )G(W)+\alpha W_E - \big ((1-\alpha )G(V)+\alpha W_E\big )\Vert \\&= (1-\alpha )\Vert G(W)-G(V)\Vert \\&\le (1-\alpha )q\,\Vert W-V\Vert . \end{aligned}$$

Because $(1-\alpha )q<1$, the map *F* is a contraction. Hence it has a unique fixed point $W^*\in \Omega$. $\square$

### Coupled convergence under the two-stage scheme

The previous two propositions naturally combine into a convergence statement for the two-stage procedure described in Section “Proposed human-centric fuzzy decision support system”.

#### Theorem 1.4 

*Assume (A1)–(A4). Suppose that at each outer iteration* *k, the inner reasoning loop is run to its fixed point* $A^*(W^{(k)})$ *, and the weight matrix is then updated by*

$$\begin{aligned} \begin{aligned} W^{(k+1)} = F(W^{(k)}). \end{aligned} \end{aligned}$$

*Then the sequence* $\{W^{(k)}\}$ *converges to the unique fixed point* $W^*$ *of F. Moreover, the corresponding equilibrium activations satisfy*

$$\begin{aligned} \begin{aligned} A^*(W^{(k)}) \longrightarrow A^*(W^*) \qquad \text {as } k\rightarrow \infty . \end{aligned} \end{aligned}$$

#### Proof

By Proposition 5.3, the map *F* is a contraction on $\Omega$, so $\{W^{(k)}\}$ converges to its unique fixed point $W^*$.

It remains to show that the equilibrium activation depends continuously on *W*. Let $W,V\in \Omega$. Since

$$\begin{aligned} \begin{aligned} A^*(W)=f(A^*(W)W), \qquad A^*(V)=f(A^*(V)V), \end{aligned} \end{aligned}$$

we estimate

$$\begin{aligned} \Vert A^*(W)-A^*(V)\Vert&\le \Vert f(A^*(W)W)-f(A^*(V)W)\Vert + \Vert f(A^*(V)W)-f(A^*(V)V)\Vert \\&\le L_f\Vert W\Vert \,\Vert A^*(W)-A^*(V)\Vert + L_f\Vert A^*(V)\Vert \,\Vert W-V\Vert . \end{aligned}$$

Since $A^*(V)\in [0,1]^n$, it is bounded in norm by a constant depending only on *n*. Using $\Vert W\Vert \le \kappa <1/L_f$, we obtain

$$\begin{aligned} \begin{aligned} (1-L_f\kappa )\Vert A^*(W)-A^*(V)\Vert \le L_f C_n \Vert W-V\Vert , \end{aligned} \end{aligned}$$

for a suitable constant $C_n>0$. Hence $W\mapsto A^*(W)$ is Lipschitz continuous on $\Omega$. Because $W^{(k)}\rightarrow W^*$, it follows that

$$\begin{aligned} \begin{aligned} A^*(W^{(k)})\rightarrow A^*(W^*). \end{aligned} \end{aligned}$$

$$\square$$

#### Remark 5.2

Theorem 5.4 is intentionally stated for the two-stage scheme in which the reasoning dynamics are stabilized before the weights are updated. This avoids the difficulty of applying a fixed-point argument directly to a fully time-varying reasoning map. The result should therefore be interpreted as a sufficient-condition analysis for the proposed training schedule, not as a universal convergence theorem for all human-in-the-loop FCM variants.

#### Example 1

*(Illustration of the Assumptions)* Consider the toy matrix in (14). If the sigmoid parameter is $\lambda =1$, then $L_f\le 1/4$. Any matrix norm satisfying $\Vert W^{(1)}\Vert <4$ guarantees that the fixed-weight inner map is contractive. This does not prove convergence of the full learning algorithm by itself; rather, it shows how the sufficient conditions in Proposition 5.1 may be checked in a simple case.

## Evaluation protocol and benchmark design

This section presents the proposed evaluation protocol for assessing the HCFDSS from four complementary perspectives: predictive performance, interpretability, sensitivity to expert intervention, and robustness under imperfect data conditions. In response to the concerns raised by the reviewers, the benchmark design is organized so that all implementation choices are explicit and reproducible.

### Experimental design

The empirical evaluation was designed to assess the proposed HCFDSS from four complementary perspectives: predictive performance, structural interpretability, sensitivity to expert intervention, and robustness under imperfect data conditions. The study includes:

1. Two synthetic medical scenarios designed to isolate nonlinear interactions and expert-correction behavior;
2. Three public medical benchmark datasets, namely the UCI Heart Disease dataset, the Breast Cancer Wisconsin Diagnostic dataset, and the Parkinsons dataset;
3. Comparisons with fixed-weight FCM, learned FCM without expert correction, logistic regression, support vector machine, random forest, gradient boosting, k-nearest neighbors, multilayer perceptron, and the proposed HCFDSS;
4. Repeated runs with statistical reporting;
5. An ablation study for the expert-correction coefficient $\alpha$;
6. Robustness analysis under missingness and class imbalance;
7. A small scalability study under expanded concept graphs.

### Synthetic datasets

Two synthetic datasets were generated. The first represents a cardiovascular-risk scenario and the second a metabolic-risk scenario. Each synthetic dataset contains $N=2000$ samples.

#### Cardiovascular scenario

The cardiovascular scenario uses the normalized feature vector

$$\begin{aligned} \begin{aligned} x=(x_1,\dots ,x_8), \end{aligned} \end{aligned}$$

where the components represent, for example, systolic pressure, chest discomfort intensity, cholesterol, heart-rate irregularity, age-related risk, smoking score, family-history score, and body-mass indicator.

A representative data-generation design is:

$$\begin{aligned} x_1&\sim \mathcal {N}(0.68,0.12^2),\\ x_2&\sim \textrm{Beta}(3,2),\\ x_3&\sim \mathcal {N}(0.62,0.10^2),\\ x_4&\sim \textrm{Beta}(2,3),\\ x_5&\sim \mathcal {N}(0.55,0.15^2),\\ x_6&\sim \textrm{Beta}(2,2),\\ x_7&\sim \textrm{Beta}(3,3),\\ x_8&\sim \mathcal {N}(0.50,0.12^2), \end{aligned}$$

with all variables truncated to [0, 1] after generation.

The ground-truth disease probability is defined by

15 $$\begin{aligned} p_{\textrm{cv}}(x) = \sigma \!\left( 1.40x_1 + 1.15x_2 + 0.90x_1x_3 + 0.55\log (1+x_4) +0.40x_6 - 1.25 \right) , \end{aligned}$$

and the binary label is sampled as

$$\begin{aligned} \begin{aligned} y\sim \textrm{Bernoulli}(p_{\textrm{cv}}(x)). \end{aligned} \end{aligned}$$

Additive Gaussian noise with standard deviation 0.03 is then applied to the observed features.

#### Metabolic scenario

The metabolic scenario uses seven normalized features associated with glucose, insulin resistance, BMI, triglycerides, activity level, age-related vulnerability, and diet-risk index. The data are generated analogously, with a nonlinear disease probability of the form

16 $$\begin{aligned} p_{\textrm{met}}(x) = \sigma \!\left( 1.30x_1 + 0.95x_2 + 0.85x_3x_4 + 0.50\log (1+x_5) -0.45x_6 + 0.35x_7 - 1.10 \right) . \end{aligned}$$

### Public benchmark datasets

To address the concern that the original evaluation used only one public dataset, the revised benchmark includes three publicly available medical datasets from the UCI Machine Learning Repository. These datasets represent different diagnostic contexts: cardiovascular disease, breast cancer diagnosis, and Parkinson’s disease detection from biomedical voice measurements (Tables 1).

**Table 1:** Public benchmark datasets used in the revised evaluation.

Dataset	Source	Instances	Features	Download link
Heart disease	UCI machine learning repository	303	13 predictive attributes	https://archive.ics.uci.edu/dataset/45/heart+disease
Breast cancer wisconsin diagnostic	UCI machine learning repository	569	30 real-valued features	https://archive.ics.uci.edu/dataset/17/breast+cancer+wisconsin+diagnostic
Parkinsons	UCI machine learning repository	195 voice recordings	22 biomedical voice features	https://archive.ics.uci.edu/dataset/174/parkinsons

For the Heart Disease dataset^21^, the common binary classification setting was used: target value 0 was treated as absence of heart disease, whereas target values 1, 2, 3, 4 were grouped as presence of heart disease. For the Breast Cancer Wisconsin Diagnostic dataset, the target labels were recoded as benign versus malignant. For the Parkinsons dataset, the target variable status was used, where 0 denotes a healthy control and 1 denotes Parkinson’s disease.

All public datasets were evaluated using the same repeated stratified train–validation–test protocol. Missing numerical variables were imputed using the training-set median, categorical variables were imputed using the training-set mode, numerical variables were standardized for scale-sensitive models, and categorical variables were one-hot encoded when required. Hyperparameters were selected using the validation set only, and final performance was reported on the held-out test partition.

### Preprocessing, fuzzification, and FCM construction

For all datasets, the data were split into training, validation, and test sets using a stratified ratio of 60:20:20. In addition, the full protocol was repeated over $R=20$ independent random seeds in order to assess variability and reduce dependence on a single random split.

For the UCI Heart Disease dataset, continuous and ordinal numerical variables were normalized by (2). The continuous variables were then represented by three Gaussian membership functions corresponding to the linguistic labels *Low*, *Medium*, and *High*. The membership centers were initialized near the lower tercile, median, and upper tercile of the normalized training distribution and then adjusted slightly for semantic plausibility. Binary and nominal categorical variables such as sex, fasting blood sugar, exercise-induced angina, and thalassemia status were encoded as discrete concept activations rather than Gaussian fuzzy variables.

Table 2 reports the final Gaussian membership parameters used for the continuous and ordinal variables in the UCI Heart Disease experiment.

**Table 2:** Gaussian membership-function parameters used for the normalized continuous and ordinal variables in the UCI Heart Disease experiment.

Feature	Low $(c,\sigma )$	Medium $(c,\sigma )$	High $(c,\sigma )$
Age	(0.20, 0.18)	(0.50, 0.18)	(0.80, 0.18)
Resting blood pressure	(0.20, 0.15)	(0.50, 0.15)	(0.80, 0.15)
Serum cholesterol	(0.20, 0.18)	(0.50, 0.18)	(0.80, 0.18)
Maximum heart rate	(0.20, 0.15)	(0.50, 0.15)	(0.80, 0.15)
ST depression (oldpeak)	(0.15, 0.12)	(0.45, 0.15)	(0.80, 0.18)
Number of major vessels	(0.10, 0.10)	(0.45, 0.15)	(0.85, 0.15)

The initial FCM topology was defined by an expert-informed mask reflecting clinically plausible interactions among cardiovascular risk factors, symptomatic concepts, intermediate ischemic burden, and the final disease concept. In particular, risk factors such as age, resting blood pressure, cholesterol, and vessel obstruction were allowed to influence intermediate burden concepts, which in turn affected the diagnostic node. This structure was kept fixed during training, while only the permitted edge weights were updated.

Two FCM baselines were used:

- *Fixed expert FCM:* Topology and weights fixed after initialization;
- *Learned FCM without expert correction:* Same topology mask, but updated only through the data-driven step.

### Reproducible HCFDSS implementation for benchmarking

To make the experimental comparison fully reproducible, the HCFDSS benchmark implementation was fixed before running the repeated-split evaluation. This subsection describes the exact feature-to-concept conversion, FCM concept list, topology mask, initial expert matrix, learning rule, expert-correction operator, output node, thresholding rule, and computation of the structural interpretability score.

#### Feature-to-concept conversion

For each dataset, every predictive variable was first converted into a normalized feature value using training-set statistics only. For a numerical feature $x_j$, min–max normalization was applied as

$$\begin{aligned} \begin{aligned} z_j=\frac{x_j-x_j^{\min }}{x_j^{\max }-x_j^{\min }}, \end{aligned} \end{aligned}$$

where $x_j^{\min }$ and $x_j^{\max }$ were computed only from the training partition and then reused for validation and test data. Missing numerical values were replaced by the training-set median before normalization. Missing categorical values were replaced by the training-set mode.

Each numerical variable was mapped into three fuzzy linguistic states:

$$\begin{aligned} \begin{aligned} \mu _{j,L}(z)=\exp \!\left( -\frac{(z-0.15)^2}{2(0.18)^2}\right) , \quad \mu _{j,M}(z)=\exp \!\left( -\frac{(z-0.50)^2}{2(0.18)^2}\right) , \quad \mu _{j,H}(z)=\exp \!\left( -\frac{(z-0.85)^2}{2(0.18)^2}\right) . \end{aligned} \end{aligned}$$

The scalar concept activation used in the FCM was chosen according to the risk orientation of the feature:

$$\begin{aligned} \begin{aligned} \phi _j(x)= {\left\{ \begin{array}{ll} \mu _{j,H}(z_j), & \text {if higher values increase the disease-risk concept}, \\ \mu _{j,L}(z_j), & \text {if lower values increase the disease-risk concept}. \end{array}\right. } \end{aligned} \end{aligned}$$

For categorical variables, categories were encoded as risk activations using smoothed positive-class rates computed on the training set:

$$\begin{aligned} \begin{aligned} \phi _j(c)= \frac{\widehat{p}(y=1\mid x_j=c)-p_j^{\min }}{p_j^{\max }-p_j^{\min }+\varepsilon _0}, \qquad \varepsilon _0=10^{-8}. \end{aligned} \end{aligned}$$

This rule ensures that all concept activations lie in [0, 1] and that larger activations consistently represent stronger support for the positive diagnostic class.

#### Concept list

For the UCI Heart Disease dataset, the FCM concept set was

$$\begin{aligned} \begin{aligned} C=\{C_1,\ldots ,C_{14}\}, \end{aligned} \end{aligned}$$

where

$$\begin{aligned} \begin{aligned} \begin{array}{ll} C_1=\text {Age risk}, & C_2=\text {Sex-related risk},\\ C_3=\text {Chest-pain risk}, & C_4=\text {Resting-blood-pressure risk},\\ C_5=\text {Cholesterol risk}, & C_6=\text {Fasting-blood-sugar risk},\\ C_7=\text {Resting-ECG risk}, & C_8=\text {Low maximum-heart-rate risk},\\ C_9=\text {Exercise-induced-angina risk}, & C_{10}=\text {ST-depression risk},\\ C_{11}=\text {ST-slope risk}, & C_{12}=\text {Major-vessel risk},\\ C_{13}=\text {Thalassemia risk}, & C_{14}=\text {Heart-disease output}. \end{array} \end{aligned} \end{aligned}$$

For the Breast Cancer Wisconsin Diagnostic dataset, the 30 measured cytological variables were treated as fuzzy diagnostic-risk concepts and one additional output node was used for malignancy. For the Parkinsons dataset, the 22 biomedical voice variables were treated as fuzzy diagnostic-risk concepts and one additional output node was used for Parkinson’s disease status.

#### Topology mask and initial expert matrix

For a dataset with *d* predictive variables, the FCM contains $n=d+1$ concepts. The first *d* concepts are feature-risk concepts and the final concept is the diagnostic output node. The topology mask $M=[m_{ij}]$ is defined by

$$\begin{aligned} \begin{aligned} m_{i,n}=1 \quad (i=1,\ldots ,d),\qquad m_{n,n}=1, \end{aligned} \end{aligned}$$

and

$$\begin{aligned} \begin{aligned} m_{ij}=0 \quad \text {otherwise}. \end{aligned} \end{aligned}$$

Thus, all feature-risk concepts are allowed to influence the diagnostic concept, and the diagnostic concept has a self-memory edge. No unsupported feature-to-feature edge is introduced. This sparse topology was used to preserve interpretability and avoid learning clinically opaque relations.

The initial expert matrix $W^{(0)}$ follows the same mask. All forbidden edges are set to zero:

$$\begin{aligned} \begin{aligned} w_{ij}^{(0)}=0 \qquad \text {whenever }m_{ij}=0. \end{aligned} \end{aligned}$$

For permitted feature-to-output edges, the initial value is computed from the training set using the absolute point-biserial association between the fuzzy concept activation and the binary target:

$$\begin{aligned} \begin{aligned} w_{i,n}^{(0)} = \min \{0.90,\max \{0.05,|\rho (\phi _i,y)|\}\}, \qquad i=1,\ldots ,d. \end{aligned} \end{aligned}$$

Because the feature activations are oriented so that larger values indicate stronger positive-class risk, these weights are nonnegative. The output self-memory edge is initialized as

$$\begin{aligned} \begin{aligned} w_{n,n}^{(0)}=0.20. \end{aligned} \end{aligned}$$

This gives a bounded, sparse, and reproducible expert-prior matrix. It should be interpreted as a benchmark prior used for reproducibility rather than as a substitute for physician elicitation in a prospective clinical study.

#### Reasoning rule, learning rule, and expert correction

For a patient sample, the initial activation vector is

$$\begin{aligned} \begin{aligned} A^{(0)}=(\phi _1,\ldots ,\phi _d,0). \end{aligned} \end{aligned}$$

The feature concepts are clamped during reasoning, while the output concept is updated iteratively. The update rule is

$$\begin{aligned} \begin{aligned} A_n^{(m+1)} = \sigma \!\left( \sum _{i=1}^{d} A_i^{(0)}w_{i,n} + A_n^{(m)}w_{n,n} + b \right) , \end{aligned} \end{aligned}$$

where

$$\begin{aligned} \begin{aligned} \sigma (t)=\frac{1}{1+\exp (-t)}. \end{aligned} \end{aligned}$$

The output node is the final diagnostic concept $C_n$. For the Heart Disease dataset, this is $C_{14}$.

The learned FCM without expert correction and the HCFDSS model minimize the regularized binary cross-entropy objective

$$\begin{aligned} \begin{aligned} J(W,b) = -\frac{1}{N}\sum _{s=1}^{N} \left[ y^{(s)}\log \hat{y}^{(s)} + (1-y^{(s)})\log (1-\hat{y}^{(s)}) \right] + \beta \Vert W\Vert _F^2, \end{aligned} \end{aligned}$$

where $\hat{y}^{(s)}$ is the final output-node activation after the reasoning iterations. The gradient is computed by truncated backpropagation through the reasoning loop. The data-driven candidate update is

$$\begin{aligned} \begin{aligned} \widetilde{W}^{(k+1)} = \operatorname {Proj}_{\Omega } \left( W^{(k)}-\eta \nabla J(W^{(k)}) \right) , \qquad \Omega =[-1,1]^{n\times n}. \end{aligned} \end{aligned}$$

The bias *b* is updated by the same gradient step but is not included in the structural interpretability score.

The reviewed edge set is

$$\begin{aligned} \begin{aligned} \mathcal {S} = \{(i,n): i=1,\ldots ,d\}\cup \{(n,n)\}. \end{aligned} \end{aligned}$$

The expert-reference matrix is the initial matrix $W^{(0)}$. After the data-driven update, the HCFDSS correction is

$$\begin{aligned} \begin{aligned} W^{(k+1)} = \operatorname {Proj}_{\Omega } \left( (1-\alpha )\widetilde{W}^{(k+1)} + \alpha W_E \right) , \end{aligned} \end{aligned}$$

where

$$\begin{aligned} \begin{aligned} W_E=W^{(0)},\qquad \alpha \in \{0.1,0.3,0.5\}. \end{aligned} \end{aligned}$$

The value of $\alpha$ is selected using the validation set. The learned FCM without expert correction is the special case $\alpha =0$.

#### Threshold selection and structural interpretability

For each train–validation–test split, the classification threshold $\tau$ is selected on the validation set by maximizing the F1-score over the grid

$$\begin{aligned} \begin{aligned} \tau \in \{0.10,0.15,0.20,\ldots ,0.90\}. \end{aligned} \end{aligned}$$

The selected threshold is then fixed and applied to the held-out test set:

$$\begin{aligned} \begin{aligned} \widehat{\textrm{class}}= {\left\{ \begin{array}{ll} 1, & \hat{y}\ge \tau , \\ 0, & \hat{y}<\tau . \end{array}\right. } \end{aligned} \end{aligned}$$

An edge is considered active if

$$\begin{aligned} \begin{aligned} |w_{ij}|>0.05. \end{aligned} \end{aligned}$$

Let $\mathcal {E}_{\textrm{active}}$ denote the set of active learned edges. The structural interpretability score is computed as

$$\begin{aligned} \begin{aligned} I_{\textrm{struct}} = \frac{ \#\{(i,j)\in \mathcal {E}_{\textrm{active}}: \operatorname {sign}(w_{ij})=\operatorname {sign}(w_{ij}^{(0)}) \} }{\#\mathcal {E}_{\textrm{active}}}. \end{aligned} \end{aligned}$$

If no edge is active, the score is reported as not applicable. Since all feature activations are oriented as diagnostic-risk concepts, sign agreement with $W^{(0)}$ measures whether the learned model preserves the semantic direction of the expert-prior map.

### Baselines

To address the need for a broader and more convincing empirical comparison, the revised benchmark includes both FCM-based baselines and mainstream machine-learning classifiers. The FCM-based baselines are included to assess the contribution of expert-guided adaptation, whereas the machine-learning baselines are included to evaluate whether the proposed framework remains competitive against commonly used predictive models.

The selected machine-learning baselines represent standard linear, kernel-based, ensemble, distance-based, neural, and neuro-fuzzy approaches, including support vector machines^22^, random forests^23^, nearest-neighbor classification^24^, and ANFIS^25^.

The comparative framework consists of the following models:

1. *Fixed expert FCM*: the expert-defined topology and initial weights are used without iterative learning;
2. *Learned FCM without expert correction*: the same topology mask is used, but weights are updated only through the data-driven learning step;
3. *Logistic regression*: a standard linear and interpretable statistical classifier;
4. *Support vector machine*: implemented with both linear and radial-basis-function kernels when applicable;
5. *Random forest*: an ensemble tree-based classifier used as a robust nonlinear baseline;
6. *Gradient boosting*: a boosted tree-based classifier used as a strong nonlinear machine-learning baseline;
7. *k-nearest neighbors*: a distance-based nonparametric classifier;
8. *Multilayer perceptron*: a feedforward neural-network classifier;
9. *HCFDSS*: the proposed human-centric adaptive FCM framework.

ANFIS is acknowledged as a relevant neuro-fuzzy comparator^25^, but it was not included in the final numerical tables because the revised benchmark focused on classifiers that could be evaluated reproducibly under the same preprocessing and validation protocol across all three datasets.

All baselines are trained and evaluated using the same train–validation–test partitions, the same preprocessing pipeline, and the same repeated stratified splits. Hyperparameters are selected using the validation set only. Test-set results are reported as mean ± standard deviation over repeated runs. This design allows the predictive performance of HCFDSS to be assessed against mainstream machine-learning methods while preserving the separate evaluation of structural interpretability, which is meaningful primarily for the FCM-based models.

**Table 3:** Validation-based hyperparameter grids used for the comparative baselines.

Model	Validation grid
Logistic regression	$C\in \{0.01,0.1,1,10\}$; class weight $\in \{\textrm{none},\textrm{balanced}\}$
SVM	Kernel $\in \{\textrm{linear},\textrm{RBF}\}$; $C\in \{0.1,1,10\}$; $\gamma \in \{\textrm{scale},0.01,0.1,1\}$
Random forest	Trees $\in \{100,300,500\}$; maximum depth $\in \{\textrm{none},3,5,10\}$
Gradient boosting	Estimators $\in \{50,100,200\}$; learning rate $\in \{0.01,0.05,0.1\}$; maximum depth $\in \{2,3,5\}$
*k*-nearest neighbors	$k\in \{3,5,7,9,11\}$; weights $\in \{\textrm{uniform},\textrm{distance}\}$
Multilayer perceptron	Hidden units $\in \{(16),(32),(16,8)\}$; regularization $\in \{10^{-4},10^{-3},10^{-2}\}$
HCFDSS	$\eta \in \{0.001,0.005,0.01\}$; $\beta \in \{10^{-4},10^{-3},10^{-2}\}$; $\alpha \in \{0.1,0.3,0.5\}$

### Implementation details

The benchmark protocol adopts the following default configuration:

$$\begin{aligned} \begin{aligned} \eta = 0.01,\qquad \beta = 10^{-3},\qquad \lambda = 1,\qquad T_{\max } = 50,\qquad \varepsilon = 10^{-4}. \end{aligned} \end{aligned}$$

The classification threshold $\tau$ is to be selected on the validation data by maximizing the F1-score. In the reference HCFDSS configuration, gradient clipping at level 1.0 is applied during the outer update, and the expert-correction coefficient is set to

$$\begin{aligned} \begin{aligned} \alpha _k = 0.3. \end{aligned} \end{aligned}$$

A validation-based tuning grid is proposed for the framework:

$$\begin{aligned} \begin{aligned} \eta \in \{0.001, 0.005, 0.01\},\qquad \beta \in \{10^{-4},10^{-3},10^{-2}\},\qquad \lambda \in \{0.5,1.0,1.5\}, \end{aligned} \end{aligned}$$

together with

$$\begin{aligned} \begin{aligned} \alpha \in \{0.1,0.3,0.5\} \quad \text {and} \quad \tau \in \{0.45,0.50,0.55,0.60\}. \end{aligned} \end{aligned}$$

The parameter ranges above define the validation-based tuning grid proposed for the benchmark protocol. In the implemented benchmark, the final configuration was selected using validation performance under repeated stratified splits. All mainstream machine-learning baselines are tuned under the same train–validation protocol to ensure fairness. The validation grids used for these baselines are summarized in Table 3. After hyperparameter selection, each model is retrained on the training partition and evaluated on the held-out test partition. The entire process is repeated over the same stratified random splits for all models.

### Validation of preprocessing choices

Because preprocessing can strongly affect performance, the effects of missing-value imputation, normalization, and fuzzification were explicitly examined.

First, missing-value handling was evaluated by comparing complete-case analysis with training-set imputation. Numerical missing values were replaced by the median computed on the training partition, while categorical missing values were replaced by the mode computed on the training partition. This prevents information from the validation or test partitions from leaking into the preprocessing stage.

Second, normalization was evaluated for scale-sensitive models. Logistic regression, SVM, *k*-nearest neighbors, multilayer perceptron, and HCFDSS were evaluated after scaling or normalization based only on the training partition. Tree-based models were also evaluated under the same split structure, but their performance was not expected to depend strongly on monotone feature scaling.

Third, fuzzification was validated for the FCM-based models by comparing direct normalized activations with Gaussian fuzzy membership activations. The fuzzy representation was retained because it provides clinically interpretable low, medium, and high concept states while preserving competitive predictive performance.

The final preprocessing pipeline was therefore selected because it avoids data leakage, improves numerical conditioning for scale-sensitive models, and produces interpretable concept activations for the FCM reasoning stage.

### Evaluation metrics

We report the following metrics:

- Accuracy;
- Precision, recall, and F1-score;
- AUROC, when applicable;
- Structural interpretability score;
- Adaptation speed;
- Stability indicator.

The structural interpretability score is defined operationally rather than impressionistically. Let $\mathcal {E}_{\textrm{active}}$ denote the set of active edges in the learned map. We define

17 $$\begin{aligned} I_{\textrm{struct}} = \frac{\#\{(i,j)\in \mathcal {E}_{\textrm{active}}:\text {sign and semantic role agree with expert prior}\}}{\#\mathcal {E}_{\textrm{active}}}. \end{aligned}$$

This score measures the proportion of active learned relations that remain semantically aligned with medically plausible expectations.

In addition, local interpretability was assessed qualitatively by checking whether the dominant activated concepts in the final equilibrium state were clinically consistent with the final classification. In particular, higher activations of chest pain severity, vessel obstruction, ST depression, and exercise-induced angina were expected to correspond to stronger support for the positive disease class.

Adaptation speed was measured by the number of outer iterations required to satisfy

$$\begin{aligned} \begin{aligned} \Vert W^{(k+1)}-W^{(k)}\Vert \le \delta , \end{aligned} \end{aligned}$$

for a prescribed tolerance $\delta =10^{-4}$. The stability indicator reports the mean norm difference between the final two weight iterates over repeated runs.

### Comparative evaluation with mainstream machine-learning baselines

The comparative evaluation is designed to determine whether the proposed HCFDSS remains predictive enough to be useful while providing stronger structural interpretability than purely data-driven alternatives. In addition to the FCM-based baselines, the revised benchmark includes mainstream machine-learning classifiers: logistic regression, support vector machine, random forest, gradient boosting, *k*-nearest neighbors, and multilayer perceptron. ANFIS is acknowledged as a relevant neuro-fuzzy comparator^25^, but it was not included in the final numerical tables because the revised benchmark focused on classifiers that could be evaluated reproducibly under the same preprocessing and validation protocol across all three datasets.

For each dataset and each repeated split, the models are trained using the same preprocessing pipeline and the same training, validation, and test partitions. Predictive performance is assessed using accuracy, precision, recall, F1-score, AUROC, and, under imbalanced settings, balanced accuracy. For FCM-based models, structural interpretability, adaptation speed, and stability are also reported. Since structural interpretability depends on concept-level edges and expert semantic agreement, this metric is not directly applicable to black-box or non-graph classifiers.

The purpose of the expanded comparison is not to claim that HCFDSS universally dominates high-capacity predictors, but to clarify its trade-off between predictive performance and clinical inspectability. In particular, a successful outcome is that HCFDSS remains competitive in classification metrics while providing editable concept-level reasoning that is unavailable in most mainstream machine-learning baselines (Tables 4, 5, 6).

**Table 4:** Comparative predictive performance on the UCI Heart Disease dataset. Results are reported as mean ± standard deviation over 20 repeated stratified train–validation–test splits.

Model	Accuracy	Precision	Recall	F1-score	AUROC	Balanced accuracy	$I_{\textrm{struct}}$
Fixed expert FCM	0.600 ± 0.045	0.536 ± 0.027	0.982 ± 0.018	0.693 ± 0.023	0.905 ± 0.030	0.629 ± 0.041	1.000 ± 0.000
Learned FCM without expert correction	0.818 ± 0.051	0.799 ± 0.088	0.830 ± 0.089	0.808 ± 0.049	0.899 ± 0.032	0.819 ± 0.049	0.865 ± 0.049
Logistic regression	0.844 ± 0.044	0.858 ± 0.062	0.796 ± 0.079	0.823 ± 0.054	0.907 ± 0.030	0.841 ± 0.045	N/A
SVM	0.826 ± 0.035	0.839 ± 0.061	0.777 ± 0.076	0.803 ± 0.044	0.899 ± 0.029	0.822 ± 0.036	N/A
Random forest	0.816 ± 0.048	0.825 ± 0.070	0.766 ± 0.064	0.792 ± 0.053	0.895 ± 0.028	0.812 ± 0.047	N/A
Gradient boosting	0.796 ± 0.031	0.804 ± 0.049	0.741 ± 0.076	0.768 ± 0.040	0.868 ± 0.040	0.792 ± 0.033	N/A
*k*-nearest neighbors	0.816 ± 0.040	0.820 ± 0.052	0.771 ± 0.068	0.793 ± 0.049	0.864 ± 0.045	0.813 ± 0.041	N/A
Multilayer perceptron	0.702 ± 0.111	0.685 ± 0.118	0.730 ± 0.133	0.695 ± 0.089	0.784 ± 0.094	0.705 ± 0.103	N/A
HCFDSS	0.811 ± 0.058	0.786 ± 0.087	0.830 ± 0.079	0.802 ± 0.054	0.905 ± 0.030	0.812 ± 0.055	1.000 ± 0.000

**Table 5:** Generalization evaluation across multiple public medical datasets. Results are reported as mean ± standard deviation over 20 repeated stratified train–validation–test splits.

Dataset	Model	Accuracy	F1-score	AUROC	Balanced accuracy
Heart disease	Logistic regression	0.844 ± 0.044	0.823 ± 0.054	0.907 ± 0.030	0.841 ± 0.045
Heart disease	SVM	0.826 ± 0.035	0.803 ± 0.044	0.899 ± 0.029	0.822 ± 0.036
Heart disease	Random forest	0.816 ± 0.048	0.792 ± 0.053	0.895 ± 0.028	0.812 ± 0.047
Heart disease	Gradient boosting	0.796 ± 0.031	0.768 ± 0.040	0.868 ± 0.040	0.792 ± 0.033
Heart disease	*k*-nearest neighbors	0.816 ± 0.040	0.793 ± 0.049	0.864 ± 0.045	0.813 ± 0.041
Heart disease	Multilayer perceptron	0.702 ± 0.111	0.695 ± 0.089	0.784 ± 0.094	0.705 ± 0.103
Breast cancer wisconsin diagnostic	Logistic regression	0.968 ± 0.016	0.955 ± 0.023	0.993 ± 0.006	0.962 ± 0.020
Breast cancer wisconsin diagnostic	SVM	0.969 ± 0.015	0.957 ± 0.022	0.994 ± 0.005	0.962 ± 0.019
Breast cancer wisconsin diagnostic	Random forest	0.955 ± 0.020	0.937 ± 0.028	0.986 ± 0.012	0.949 ± 0.024
Breast cancer wisconsin diagnostic	Gradient boosting	0.955 ± 0.020	0.937 ± 0.029	0.989 ± 0.011	0.947 ± 0.024
Breast cancer wisconsin diagnostic	*k*-nearest neighbors	0.960 ± 0.018	0.944 ± 0.025	0.982 ± 0.012	0.951 ± 0.021
Breast cancer wisconsin diagnostic	Multilayer perceptron	0.904 ± 0.046	0.871 ± 0.059	0.967 ± 0.023	0.897 ± 0.044
Parkinsons	Logistic regression	0.858 ± 0.058	0.904 ± 0.041	0.916 ± 0.042	0.813 ± 0.079
Parkinsons	SVM	0.915 ± 0.046	0.944 ± 0.029	0.949 ± 0.050	0.876 ± 0.078
Parkinsons	Random forest	0.917 ± 0.037	0.945 ± 0.025	0.964 ± 0.023	0.872 ± 0.055
Parkinsons	Gradient boosting	0.914 ± 0.040	0.944 ± 0.025	0.963 ± 0.036	0.860 ± 0.076
Parkinsons	*k*-nearest neighbors	0.891 ± 0.045	0.928 ± 0.029	0.962 ± 0.027	0.845 ± 0.081
Parkinsons	Multilayer perceptron	0.727 ± 0.079	0.826 ± 0.056	0.618 ± 0.200	0.581 ± 0.121

**Table 6:** Cross-dataset performance of FCM-based models. Results are reported as mean ± standard deviation over 20 repeated stratified train–validation–test splits.

Dataset	Model	Accuracy	F1-score	AUROC	Balanced accuracy	$I_{\textrm{struct}}$
Heart disease	Fixed expert FCM	0.600 ± 0.045	0.693 ± 0.023	0.905 ± 0.030	0.629 ± 0.041	1.000 ± 0.000
Heart disease	Learned FCM without expert correction	0.818 ± 0.051	0.808 ± 0.049	0.899 ± 0.032	0.819 ± 0.049	0.865 ± 0.049
Heart disease	HCFDSS	0.811 ± 0.058	0.802 ± 0.054	0.905 ± 0.030	0.812 ± 0.055	1.000 ± 0.000
Breast cancer wisconsin diagnostic	Fixed expert FCM	0.867 ± 0.056	0.830 ± 0.050	0.964 ± 0.014	0.865 ± 0.038	1.000 ± 0.000
Breast cancer wisconsin diagnostic	Learned FCM without expert correction	0.882 ± 0.027	0.840 ± 0.032	0.955 ± 0.013	0.872 ± 0.024	0.889 ± 0.033
Breast cancer wisconsin diagnostic	HCFDSS	0.871 ± 0.042	0.833 ± 0.045	0.964 ± 0.014	0.871 ± 0.034	1.000 ± 0.000
Parkinsons	Fixed expert FCM	0.744 ± 0.000	0.853 ± 0.000	0.848 ± 0.071	0.500 ± 0.000	1.000 ± 0.000
Parkinsons	Learned FCM without expert correction	0.749 ± 0.024	0.851 ± 0.018	0.824 ± 0.066	0.538 ± 0.077	0.968 ± 0.030
Parkinsons	HCFDSS	0.818 ± 0.044	0.889 ± 0.025	0.848 ± 0.070	0.663 ± 0.082	1.000 ± 0.000

The expanded benchmark gives a more balanced view of the proposed framework. On the UCI Heart Disease dataset, logistic regression achieved the highest accuracy and F1-score among the tested models, with accuracy $0.844\pm 0.044$ and F1-score $0.823\pm 0.054$. The proposed HCFDSS achieved accuracy $0.811\pm 0.058$, F1-score $0.802\pm 0.054$, and AUROC $0.905\pm 0.030$, which is close to the AUROC of logistic regression $0.907\pm 0.030$. Thus, HCFDSS is competitive on the cardiovascular benchmark but is not claimed to dominate all mainstream machine-learning models in raw predictive performance.

The FCM-based comparison clarifies the effect of expert correction. The fixed expert FCM achieved very high recall but low precision, indicating a tendency to over-predict the positive class. The learned FCM without expert correction improved accuracy and F1-score, but its structural interpretability score decreased to $0.865\pm 0.049$. By contrast, HCFDSS preserved full structural sign consistency with the expert-prior map, with $I_{\textrm{struct}}=1.000\pm 0.000$, while maintaining comparable predictive performance. This supports the intended role of HCFDSS as an interpretable and expert-editable decision-support framework.

The additional Breast Cancer Wisconsin Diagnostic and Parkinsons datasets evaluate generalization beyond the original cardiovascular setting. On Breast Cancer Wisconsin Diagnostic, mainstream classifiers such as SVM and logistic regression achieved stronger predictive performance than HCFDSS, reflecting the advantage of standard classifiers on high-dimensional cytological feature data. On Parkinsons, HCFDSS improved over the fixed and purely learned FCM variants in balanced accuracy while preserving structural interpretability. Overall, the revised results show that HCFDSS should be interpreted not as a universal replacement for high-capacity predictors, but as a transparent concept-level model that trades some predictive flexibility for editability, semantic consistency, and structural interpretability.

### Sensitivity to the expert-correction coefficient

To examine the human-centric component explicitly, the benchmark protocol varies

$$\begin{aligned} \begin{aligned} \alpha \in \{0,0.1,0.3,0.5,0.7,0.9\}. \end{aligned} \end{aligned}$$

The case $\alpha =0$ corresponds to purely data-driven learning without expert correction. The purpose of this ablation is to assess how the level of expert intervention influences predictive quality, structural interpretability, and convergence speed.

In a completed empirical study, the sensitivity analysis should report the corresponding performance metrics across repeated runs and should clarify whether moderate values of $\alpha$ provide a better balance between semantic preservation and data-driven flexibility. The intended interpretation is that very small values of $\alpha$ may underuse expert knowledge, whereas values too close to one may overconstrain the adaptation process.

### Robustness under missingness and imbalance

To test robustness, we introduced missingness rates of $10\%$, $20\%$, and $30\%$ by randomly masking observed features under a missing-completely-at-random protocol. Missing numerical values were imputed by the median computed on the training set, while categorical values were imputed by the mode computed on the training set. This protocol was applied consistently across all models.

To evaluate class imbalance, additional training subsets were created with class ratios of 1 : 1, 1 : 2, and 1 : 3 by controlled downsampling of the positive class in the training partition only. The standard objective (7) was then compared with the cost-sensitive objective (9), using class-dependent penalties

$$\begin{aligned} \begin{aligned} (\omega _0,\omega _1)\in \{(1,1),(1,2),(1,3)\}. \end{aligned} \end{aligned}$$

The robustness analysis is included in the benchmark design in order to assess not only predictive degradation but also whether the proposed framework preserves interpretability under increasingly imperfect data conditions. In a completed implementation, the corresponding results should be reported under each missingness level and imbalance condition using the same repeated validation protocol as the main benchmark.

### Scalability analysis

Scalability is assessed by increasing the number of concepts in the FCM while preserving sparse expert-informed topology masks. Starting from the base cardiovascular concept graph, expanded graphs are considered with

$$\begin{aligned} \begin{aligned} n \in \{12,20,30\} \end{aligned} \end{aligned}$$

concepts and approximate edge density 0.20. For each graph size, the intended empirical quantities are the average runtime per outer iteration, the total number of outer iterations required to satisfy the stopping criterion, and the final stability indicator.

From a computational perspective, the dominant cost of one inner reasoning iteration is the matrix-vector multiplication in (4), which scales as $O(n^2)$ for dense weight matrices and more favorably under sparse topology masks. For a training set of size *N*, $T_{\max }$ inner reasoning iterations, and $K_{\max }$ outer updates, the overall cost is of order

$$\begin{aligned} \begin{aligned} O(K_{\max } N T_{\max } n^2), \end{aligned} \end{aligned}$$

up to implementation-dependent constants and sparsity effects. The expert-correction step acts only on the reviewed edge set $\mathcal {S}_k$, and therefore adds at most $O(|\mathcal {S}_k|)$ per outer iteration.

This subsection is therefore intended to clarify both the empirical benchmarking protocol and the expected scaling behavior of the proposed HCFDSS as the concept graph becomes richer.

## Discussion

The revised study should be interpreted in light of its actual scope. The proposed HCFDSS is not intended to replace all high-capacity medical predictors, nor does it claim to infer causal truth directly from observational data. Its main purpose is to provide a transparent concept-level decision-support mechanism in which data-driven adaptation can be moderated by structured expert intervention.

### What the proposed framework adds

The main practical value of HCFDSS lies in the combination of three properties that are rarely present simultaneously: concept-level interpretability, editable adaptation, and explicit separation between reasoning and learning. Compared with a fixed expert-defined FCM, the proposed model can respond to new evidence. Compared with purely data-driven FCM learning, it offers a mechanism for preserving semantic structure through bounded expert correction. Compared with black-box models, it remains inspectable in terms of named concepts and directed relations, although the expanded benchmark shows that standard machine-learning models may achieve higher raw predictive performance on some datasets.

For this reason, the most appropriate comparison is not with every state-of-the-art deep model on raw predictive performance, but with methods intended for structured, interpretable, and clinically reviewable decision support. Compared with mainstream interpretable deep-learning approaches, the core competitive advantage of HCFDSS is that the learned reasoning structure remains editable at the concept and edge level, allowing direct expert correction of clinically meaningful relations rather than only post hoc explanation of predictions.

### Interpretability beyond a single ratio

A recurring weakness in the early version of the manuscript was that interpretability was described too loosely. In the revised framework, interpretability is treated at two levels. First, structural interpretability measures whether the learned active relations remain consistent with clinically acceptable semantics. Second, local interpretability examines whether the dominant concepts in the equilibrium state align with plausible medical reasoning for individual cases.

This distinction is important. A model may retain a sparse graph while still producing equilibrium activations that do not make clinical sense. Conversely, a model may fit the labels well while losing concept-level meaning. A useful medical decision-support system must control both aspects.

### Expert intervention as a controlled rather than absolute mechanism

Another important point is that expert intervention should not be modeled as an unquestionable override. In the present formulation, disagreement between the data-driven update and expert judgment is handled through the convex blending rule (12). This makes the intervention interpretable and stable while still allowing the model to benefit from the information present in the data.

The sensitivity study in $\alpha$ is therefore central rather than auxiliary. Small values of $\alpha$ may underuse clinical expertise, whereas values too close to one may suppress useful data-driven correction. The purpose of the analysis is not to claim a universal optimal value, but to show how the expert-intervention level affects the balance between fit, interpretability, and convergence.

### Missingness, imbalance, and clinical cost sensitivity

Real clinical data are often incomplete and imbalanced. For that reason, a purely synthetic evaluation is not enough, and the present work therefore includes robustness analysis and a cost-sensitive extension as part of the proposed benchmark protocol. This is especially important in diagnosis, where false negatives and false positives can carry very different clinical consequences. The weighted objective in (9) is a natural way to account for this asymmetry without changing the overall structure of the framework.

### Scalability and limitations

The framework also has limitations. As the number of concepts increases, expert elicitation becomes harder, the number of candidate edges grows rapidly, and maintaining stable reasoning dynamics becomes more challenging. Sparse topology constraints, modular decomposition, and disease-specific concept grouping are likely to be important in larger clinical models.

A second limitation is theoretical. The convergence result in Section “Theoretical analysis” is established under explicit assumptions, including strong convexity of the outer objective and boundedness of the admissible weight set. These assumptions make the analysis clear, but they may not describe every practical implementation.

A third limitation is empirical. Although the revised study now includes three public benchmark datasets, the results show that HCFDSS is not uniformly superior to mainstream machine-learning models in raw predictive performance. Its main advantage lies in structural interpretability, semantic preservation, and expert editability. Therefore, the present study should still be viewed as a methodological paper with medical decision-support motivation, not as a clinically validated diagnostic tool. Prospective clinical studies and physician-centered usability evaluations remain outside the scope of the current manuscript.

### Practical implication

Despite these limitations, the revised framework is promising in settings where clinicians want to inspect and refine the internal logic of the decision-support model. The emphasis on concept-level reasoning, bounded correction, and transparent structure makes HCFDSS especially suitable for collaborative human–AI workflows in which the model is expected to remain interpretable throughout its adaptation process.

## Conclusion

This paper studied a human-centric adaptive framework for fuzzy cognitive map-based medical decision support. The proposed HCFDSS combines fuzzy concept modeling, bounded FCM reasoning, regularized weight learning, and an explicit expert-correction operator. The main methodological contribution is not the claim of a universally new hybrid rule, but a clear and interpretable formulation in which expert intervention is integrated into the update mechanism in a controlled manner.

To support this framework mathematically, the analysis was organized into two stages: a fixed-weight reasoning stage and an outer weight-update stage. Under standard smoothness and boundedness assumptions, sufficient conditions were derived for the inner fixed-point dynamics and the convergence of the projected expert-corrected outer update. This yields a more careful theoretical justification than treating the entire adaptive process as a single time-varying contraction.

From an empirical perspective, the paper reports a concrete benchmarking study involving synthetic scenarios, three public medical datasets, expanded mainstream machine-learning comparisons, sensitivity analysis with respect to expert intervention, preprocessing validation, and robustness checks under missingness and imbalance. This protocol is intended to clarify how the proposed framework should be assessed in terms of predictive performance, interpretability, and adaptability under controlled and reproducible conditions.

Overall, the HCFDSS framework should be viewed as a transparent and editable decision-support model rather than a replacement for all high-capacity medical predictors. Its value lies in enabling clinicians to inspect, question, and refine concept-level relations while still allowing the model to adapt. Future work should include broader clinical validation studies, physician-centered usability evaluation, temporal extensions, and richer uncertainty models such as interval or type-2 fuzzy cognitive maps.

## Data Availability

The synthetic datasets described in Section “Synthetic datasets” are fully specified by the distributions and parameters reported in the manuscript and can be generated accordingly. The public benchmark datasets used in the revised evaluation are available from the UCI Machine Learning Repository: the Heart Disease dataset at https://archive.ics.uci.edu/dataset/45/heart+disease, the Breast Cancer Wisconsin Diagnostic dataset at https://archive.ics.uci.edu/dataset/17/breast+cancer+wisconsin+diagnostic, and the Parkinsons dataset at https://archive.ics.uci.edu/dataset/174/parkinsons.

## Code availability

The Python implementation used to generate the benchmark tables, including preprocessing, repeated stratified splitting, baseline training, and the reproducible HCFDSS benchmark implementation, is provided as Supplementary File 1 and is also available from the author upon reasonable request.
